# The outcomes of midline versus medio-lateral episiotomy

**DOI:** 10.1186/1742-4755-4-10

**Published:** 2007-10-29

**Authors:** Ratchadawan Sooklim, Jadsada Thinkhamrop, Pisake Lumbiganon, Witoon Prasertcharoensuk, Jeerichuda Pattamadilok, Kanok Seekorn, Chompilas Chongsomchai, Prakai Pitak, Sukanya Chansamak

**Affiliations:** 1 Department of Obstetrics and Gynecology, Faculty of Medicine, Khon Kaen University, Khon Kaen, Thailand; 2 Community Medicine Service Section, Faculty of Medicine, Khon Kaen University, Khon Kaen, Thailand; 3 Nursing Section, Faculty of Medicine, Khon Kaen University, Khon Kaen, Thailand

## Abstract

**Background:**

Episiotomy is the surgical enlargement of the vaginal orifice by an incision of the perineum during the second stage of labor or just before delivery of the baby. During the 1970s, it was common to perform an episiotomy for almost all women having their first delivery, ostensibly for prevention of severe perineum tears and easier subsequent repair. However, there are no data available to indicate if an episiotomy should be midline or medio-lateral. We compared midline versus medio-lateral episiotomy for complication such as extended perineal tears, pain scores, wound infection rates and other complications.

**Methods:**

We conducted a prospective cohort including 1,302 women, who gave birth vaginally between April 2005 and February 2006 at Srinagarind Hospital – a tertiary care center in Northeast Thailand. All women included had low risk pregnancies and delivered at term. The outcome measures included deep perineal tears (including perineal tears with anal sphincter and/or rectum tears), other complications, and women's satisfaction at 48 hours and 6-weeks postpartum.

**Results:**

In women with midline episiotomy, deep perineal tears occurred in 14.8%, which is statistically significantly higher compared to 7% in women who underwent a medio-lateral episiotomy (p-value < 0.05). There was no difference between the groups for other outcomes (such as blood loss, vaginal hematoma, infection, pain, dyspareunia, and women's satisfaction with the method). The risk factors for deep perineal tears were: midline episiotomy, primiparity, maternal height < 145 cm, fetal birth weight > 3,500 g and forceps extraction.

**Conclusion:**

Midline compared to medio-lateral episiotomy resulted in more deep perineal tears. It is more likely deep perineal tears would occur in cases with additional risk factors.

## Background

Episiotomy is the surgical enlargement of the vaginal orifice by an incision of the perineum during the second stage of labor or just before delivery and requires repair by suturing [1]. During the 1970s, episiotomies were performed on almost all women having their first delivery in order to prevent severe perineal tears and make repair easier. Another commonly cited but unproven benefit of routine episiotomy was the prevention of pelvic relaxation. A number of observational studies and randomized trials showed that routine episiotomy is associated with an increased incidence of anal sphincter and rectal tears [2].

In the Cochrane Database of systematic reviews 1999, restrictive rather than routine use of episiotomy was recommended; however, no indications were given vis-à-vis which type should be used and when, i.e., for assisted vaginal delivery (forceps or vacuum), preterm delivery, breech delivery, predicted macrosomia or a presumed imminent tear. However, we could not identify a clinical trial comparing medio-lateral versus midline episiotomy [3]. At Srinagarind Hospital, our personnel are encouraged to use restrictive episiotomy and to select the type appropriate for each case.

We aimed to evaluate midline versus medio-lateral episiotomy for deep perineal tears, pain scores, wound infection rate and other complications at 48-hours and 6-weeks postpartum.

## Methods

This was a prospective cohort study for which we recruited 1,302 pregnant women who gave birth vaginally at Srinagarind Hospital, Khon Kaen University – a tertiary care center in Northeast Thailand – between April 2005 and February 2006. These women had low risk pregnancies according to the following criteria: singleton, cephalic presentation and term pregnancy. All women received episiotomy, either midline or medio-lateral according to attending personnel. Women with spontaneous perineal tears, epidural analgesia and/or any underlying disease (such as diabetes mellitus, chronic renal disease or immune deficiency related diseases) were excluded.

We collected some of the study data from the medical records. The primary outcome was deep perineal tear, defined as a tear with anal sphincter and/or rectum tearing and assessed by the attending physician(s) or nurse(s). The amount of blood loss was estimated by the delivery attendants, based on visual inspection.

Forty-eight hours postpartum the women were asked to complete a pain scoring form after having signed informed consent. We used pain scoring scale of 0 to 10 for 'none' to 'severe' pain regarding the episiotomy wound. Six weeks postpartum, women were interviewed either during the postpartum visit or by telephone for assessment of perineal pain, wound infection, dyspareunia and their satisfaction with the procedure.

We used STATA 9.0 software for Windows (STATA Corp., College Station, TX) for data processing and analysis. Results were reported as means, standard deviations (SD), medians, ranges, relative risks (RR) and 95% confidence intervals (CI).

This study was approved by the Human Research Ethics Committee of Khon Kaen University.

## Results

Of the 1,302 pregnant women studied, 426 received midline and 876 medio-lateral episiotomy. The baseline characteristics of the two groups are shown in terms of maternal age, weight before delivery, height, parity, gestational age, duration of second stage of labour, fetal birth weight, birth asphyxia, perineal repairing time, amount of blood loss, type of vaginal delivery, type of personnel who performed the delivery and use of antibiotic prophylaxis (Table 1).

**Table 1:** Baseline characteristic of the pregnant women studied

**Characteristics**	**Midline episiotomy **(n = 426)	**Medio-lateral episiotomy **(n = 876)
Age (yr, median, min-max)	28 (16–44)	26 (12–47)
Weight before delivery (kg, median, range)	64 (44.3–93.2)	64.8 (45.4–96.3)
Height (cm, mean ± SD)	156.7 ± 5.0	156. ± 5.2
Gravidity (No., mean ± SD))	1.9 ± 0.9	1.7 ± 0.9
Nulliparous (No., %)	206 (48.4)	488 (55.7)
Multiparous (No., %)	220 (51.6)	388 (44.3)
Gestational age (wks, mean ± SD)	38.7 ± 1.3	38.9 ± 1.2
Duration of 2^nd ^stage (min, median, min-max)	13 (1–111)	14 (1–140)
Baby birth weight (gm, mean ± SD)	3,104.4 ± 346.5	3,147 ± 383.9
Birth asphyxia. (No., %) (Apgar score at one minute ≤ 7)	14(3.3)	64(7.3)
Suture's time (min, mean ± SD)	22.75 ± 11.40	26.15 ± 12.89
Estimate blood loss (ml, median, min-max)	200 (50–1,000)	200 (50–900)
Type of delivery. (No., %)		
1. normal delivery	379 (88.9)	734(84.8)
2. forceps extraction	22 (5.7)	49(5.6)
3. vacuum extraction	25 (5.9)	84(9.6)
Delivery performed by (No., %)		
1. intern	5(1.2)	57(6.5)
2. resident	155(36.4)	725(82.8)
3. staff	266(62.4)	94(10.7)
Suture material. (No., %)		
1. polyglycolic acid	28(6.6)	57(6.5)
2. non polyglycolic acid	369(86.7)	816(93.2)
3. combined	29(6.8)	3(0.3)
Antibiotic prophylaxis at LR (No., %)	28(6.6)	44(5.1)

Deep perineal tears occurred in 63 of the 426 women with midline episiotomy (14.79%), and in 61 of 876 women with medio-lateral episiotomy (6.97%) (p-value < 0.05). There was no statistically significant difference in blood loss (median 200 ml, range 50–100 ml in the midline versus median 200 ml, range 50–900 ml in the medio-lateral group) and vaginal hematoma complications after delivery (2/426 for midline versus 1/876 for medio-lateral episiotomy).

We were able to assess pain scores and wound infection 48 hours postpartum for 222 of 426 women with midline and 536 of 876 women with medio-lateral episiotomies. There was no difference in pain scores at 48 hours postpartum between the two groups (mean 3, range 0–8 in the midline versus mean 3, range 0–9 in the medio-lateral group). One case with wound infection occurred in the medio-lateral episiotomy group.

Among the women studied, we were able to contact with 312 women (24.0%) 6-weeks postpartum to assess outcomes. We found no statistically significant difference between the two groups, regarding pain scores (median 0, range 0–1 in the midline versus Median 0, range 0–5 in the medio-lateral group, p-value 0.13) and satisfaction with the episiotomy procedure (100% in the midline versus 99.1% in the medio-lateral group). There were five women with midline and 19 with medio-lateral episiotomies that had sexual intercourse before six weeks postpartum. There was no difference in dyspareunia between the two groups (0/5 in the midline versus 3/19 in the medio-lateral group) and none of them had any wound infection.

The following risk factors were associated with deep perineal tear: (1) underwent a midline episiotomy (RR 2.12; 95% CI 1.52 to 2.96); (2) primiparity (RR 3.47; 95% CI 2.27, to 5.31); (3) maternal height ≤ 145 cm (RR 2.60; 95% CI 1.19 to 5.67); (4) vacuum extraction (RR 1.92; 95% CI 1.17 to 3.15); (5) forceps extraction (RR 4.04; 95% CI 2.70 to 6.04); and, (6) duration of second stage of labour > 60 minutes (RR 2.30; 95% CI 1.17 to 4.54). We controlled for confounders of the factors affecting deep perineal tears by using a multivariate analysis. The only statistically significant factors were: (1) midline episiotomy (RR 1.94; 95% CI 1.25 to 2.99); (2) primiparity (RR 3.56; 95% CI 2.23 to 5.69); (3) maternal height ≤ 145 cm (RR 4.22; 95% CI 2.01 to 8.44); (4) fetal birth weight > 3500 g (RR 2.22; 95% CI 1.46 to 3.38); and, (5) forceps extraction (RR 2.82; 95% CI 1.89 to 4.19) (Table 2).

**Table 2:** Risk factors of deep perineal tear

**Risk factors**	**Univariate analysis RR (95% CI)**	**Multivariate analysis RR (95% CI)**
Midline episiotomy	2.12 (1.52, 2.96)*	1.94 (1.25, 2.99)*
Maternal age > 35 yrs., < 20 yrs.	0.81 (0.49, 1.33)	0.94 (0.56, 1.59)
Primiparity	3.47 (2.27, 5.31)*	3.56 (2.23, 5.69)*
Gestational age > 41 wks.	0 (α, α)	8.39 (0, α)
Maternal height ≤ 145 cm.	2.60 (1.19, 5.67)*	4.22 (2.01, 8.44)*
Maternal body mass index > 25	0.94 (0.65, 1.36)	1.01 (0.96, 1.06)
Baby birth weight > 3500 gm.	1.51 (0.99, 2.27)	2.22 (1.46, 3.38)*
Experienced Physician		
1. intern	2.50 (0, α)	9.45 (0, α)
2. resident	0.48 (0.34, 0.67)	0.69 (0.44, 1.08)
3. staff	1	1
Operative vaginal delivery		
1. vacuum extraction	1.92 (1.17, 3.15)*	1.12 (0.62, 2.00)
2. forceps extraction	4.04 (2.70, 6.04)*	2.82 (1.89, 4.19)*
3. normal vg. Delivery	1	1
Duration of second stage > 60 min	2.30 (1.17, 4.54)*	1.69 (0.81, 3.54)

*statistically significant

## Discussion

In this cohort study, 1,302 low risk pregnant women were studied for outcomes related to midline and medio-lateral episiotomies. We found that midline episiotomy resulted in a greater rate of deep perineal tears than medio-lateral episiotomies (14.8% versus 7 %) but there was no difference between the two groups on other outcomes such as blood loss, hematoma, infection, pain and dyspareunia.

The results of our study are comparable to previous reports regarding deep perineal tears; specifically, Aytan *et al*. found severe perineal lacerations in 3% of midline versus 1% of medio-lateral episiotomies [4] and Angioli *et al*. reported 6.6% in midline versus. 4.1% in medio-lateral episiotomies [5].

We found that the risk factors of deep perineal tears were midline episiotomy, primiparity, maternal height ≤ 145 cm, baby's birth weight > 3500 g and forceps extraction. Again, our results are similar to those from previous studies [6-13].

Interestingly, Werner *et al*. reported that midline episiotomy actually had less hematoma formation and blood loss [12] while we found that vaginal hematoma was the only complication which occurred in the two groups (not statistically different between groups). This is a rare complication and our study might not have had the power to detect the difference.

Coats *et al*. compared midline and medio-lateral episiotomies in a randomized controlled trial and found no difference in perineal pain immediately and 3 months postpartum [9], comparable to our results. By comparison, Werner *et al*. found significantly less pain after midline than medio-lateral episiotomies on the third day postpartum [12]. A limitation to our study might be pain assessment since the proportion of women who gave informed consent and completed the pain scoring form 48 hours postpartum was low and most of our results were drawn from telephone interviews 6-weeks postpartum.

Our study found only one case of wound infection in the medio-lateral episiotomy group at 48 hours which was absent at 6-weeks postpartum. A previous study by Larsson *et al*. assessed perineal problems after episiotomy versus spontaneous perineal laceration and found a significantly higher rate of infection in the episiotomy group [14]. But like our results, Harrison *et al*. found no case of infection for the first four days after delivery or at the 6 week postpartum check-up [15]. By way of corroboration, Owen and Hauth retrospectively reviewed women who had given vaginal birth at the University of Alabama Hospitals and found only ten cases of postpartum perineum infection among 20,713 deliveries, with all of the infectious complications occurring after midline episiotomy [16].

We found that almost all women in both groups were satisfied with their perineum scar. Fewer women in the midline group complained about dyspareunia compared to the medio-lateral group (0 versus 15.8%, respectively); however, we cannot absolutely conclude that one is better due to the small number of participants who had sexual intercourse within the 6-weeks postpartum period. A previous report indicated midline incision was preferable to medio-lateral episiotomy vis-à-vis sexual function, healing, and improved appearance of the perineal scar [17]. However, in our study, we cannot make strong conclusions regarding long-term effects due to the high rate of loss-to-follow-up. Furthermore, in our study, there was no difference on the effect of episiotomy type on blood loss. Theoretically, the line of incision in a midline episiotomy stays within an area where the muscles of the perineum from both sides connect, which should limit blood loss [12]. Additionally, we may have underestimated blood loss by depending on visual inspection instead of using a more objective measuring method. However, we may need randomized controlled trial study to assess the accuracy of the method to estimate blood loss.

Our study may not have been able to account for all possible factors, with a randomized controlled trial being the preferred study design to compare different interventions.

## Conclusion

In this cohort study we would conclude that midline episiotomies resulted in more cases of deep perineal tear compare to medio-lateral episiotomies. Further, deep perineal tears may occur more frequently in cases with additional risk factors. Although a randomized controlled trial (RCT) is the preferred study design to compare different interventions, we were unable to conduct a RCT in our setting. However, the results of our cohort study can pave the way to conduct a well-designed RCT, comparing midline versus medio-lateral episiotomy.

## Competing interests

The author(s) declare that they have no competing interests.

## Authors' contributions

Sooklim R and Thinkhamrop J made substantial contributions to the conception and design, acquisition of data, analysis and interpretation of data; Sooklim R, Pitak P and Chansamak S had contribution on data collection; Sooklim R, Thinkhamrop J, Lumbiganon P, Prasertcharoensuk W, Pattamadilok J, Chongsomchai C and Seejorn K were involved in the drafting and revising of the manuscript; Thinkhamrop J and Seejorn K finalized the version to be published.
